# Altitudinal Variation in Woody Species Diversity, Regeneration, and Soil Seed Bank Composition in Bela Mountain Forest, Northern Ethiopia

**DOI:** 10.1002/ece3.74102

**Published:** 2026-07-23

**Authors:** Abebe Shumie, Yekoye Alene, Geta Mesegan, Sileshi Asmare, Getu Abebe, Mubarek Eshetie, Menale Wondie

**Affiliations:** ^1^ Amhara Agricultural Research Institute Sekota Dryland Agricultural Research Center Sekota Ethiopia; ^2^ Amhara Agricultural Research Institute Gondar Agricultural Research Center Gondar Ethiopia; ^3^ College of Agriculture and Environmental Sciences University of Gondar Gondar Ethiopia; ^4^ Amhara Agricultural Research Institute Bahir Dar Ethiopia

**Keywords:** Altitudinal gradients, Regeneration status, Soil seed bank, Species diversity

## Abstract

Understanding the woody species distribution along altitudinal gradients is crucial for effective forest management and conservation practices. Therefore, this study aimed to examine the relationship between species diversity and regeneration status along altitudinal gradients in the Bela Mountain Forest, northern Ethiopia. A systematic sampling method was used to collect vegetation data from 123 plots measuring 20 m × 20 m, positioned at 300 m intervals along the transect lines. A total of 54 woody species were recorded, with species richness declining markedly with increasing altitude. The lower altitude had the highest number of species (35), followed by the middle (24) and higher (13) altitudes. The lower altitude also exhibited the highest mean Shannon diversity index (2.37 ± 0.20) and species richness (18.9 ± 1.06), whereas the middle altitude had the highest woody species density (466 ± 35.5 ind./ha). Regeneration declined significantly with increasing altitude, indicating reduced recruitment at higher elevations. The soil seed bank comprised 68 species belonging to 22 families, with herbaceous species representing 61.8% of the total flora. Overall soil seed bank density reached 15,141 seedlings/m^2^, suggesting substantial regeneration potential but limited representation of woody species. Altitude strongly shapes woody species diversity, regeneration dynamics, and soil seed bank composition in Bela Mountain Forest. The predominance of herbaceous species in the soil seed bank and the reduced regeneration of woody species at higher elevations indicate that natural regeneration alone may be insufficient to maintain forest structure. The findings suggest that conservation and restoration efforts should prioritize improving woody species regeneration and protecting existing mature trees to enhance long‐term forest resilience and biodiversity conservation.

## Graphical abstract

The study aimed to examine the relationship between species diversity and regeneration status along altitudinal gradients in a Bela Mountain Forest, northern Ethiopia. The result of the study showed that 54 woody species belonging to 31 families were recorded. Altitude has a considerable impact on both species diversity and richness, with the Shannon diversity index and richness highest at lower and medium altitudes.

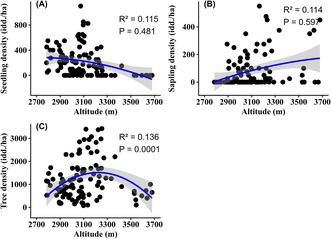

## 1 Introduction

Mountain ecosystems cover approximately 27% of the Earth's terrestrial surface and provide essential ecosystem services while supporting exceptionally high levels of biodiversity and endemism (Pandey et al. 2018; Korner and Spehn 2024). Their complex topography generates pronounced environmental heterogeneity through variation in altitude, climate, slope, and aspect, creating diverse microclimates and habitats over relatively short spatial distances (Antonelli et al. 2018; Pandey et al. 2018). Consequently, mountain ecosystems are shaped by interactions of both abiotic and biotic factors, including temperature, precipitation, soil properties, solar radiation, and species interactions (Yirga et al. 2019). Among these drivers, altitude is particularly important because it indirectly regulates environmental conditions such as temperature, moisture availability, soil characteristics, and solar exposure, thereby influencing vegetation structure, composition, and ecosystem functioning (Wani and Pant 2023; Negi et al. 2024). Together, these factors determine the distribution and diversity of mountain forest communities (Jimenez‐Paz et al. 2021; He et al. 2023).

Altitudinal gradients have long been recognized as natural laboratories for investigating the ecological processes governing plant community assembly and biodiversity patterns (Gebrehiwot et al. 2019). Changes in temperature, atmospheric pressure, soil conditions, and water availability with increasing elevation strongly influence species composition and forest structure (Negi et al. 2024; Liu et al. 2025). However, the relationship between altitude and plant diversity remains inconsistent across mountain systems. While several studies have reported a continuous decline in species richness with increasing elevation (Wani et al. 2023; Negi et al. 2024; Yimer and Atsbha 2025), others have observed a hump‐shaped pattern with maximum diversity at intermediate elevations (Che et al. 2025; Li et al. 2025). These contrasting patterns suggest that altitudinal biodiversity is regulated not only by elevation itself but also by the complex interactions among climatic conditions, edaphic properties, topography, disturbance regimes, and species interactions (Yirga et al. 2019; Gillani et al. 2025).

Understanding how woody plant communities respond to altitudinal variation is fundamental for developing effective forest conservation and sustainable management strategies (Zhang et al. 2024). Ethiopia is one of Africa's biodiversity‐rich countries, where remarkable geological and topographic heterogeneity has created diverse agroecological zones and a high level of plant diversity, including numerous endemic species (Asefa et al. 2020). Although several studies have investigated woody species diversity and distribution along altitudinal gradients in different Ethiopian mountain forests (Gebrehiwot et al. 2019; Bogale et al. 2023; Abrha et al. 2024; Yimer and Atsbha 2025), the observed patterns vary considerably among regions owing to differences in environmental conditions, vegetation history, and human disturbance (Berihun Tenaw et al. 2024). Consequently, additional studies from underrepresented mountain ecosystems are needed to improve our understanding of the ecological drivers of species distribution and to support evidence‐based forest conservation across the country.

The Bela Mountain Forest in northern Ethiopia represents one such understudied montane ecosystem. The forest encompasses a wide altitudinal range and is characterized by rugged terrain, heterogeneous habitats, and predominantly indigenous woody vegetation, including *Erica arborea* , *Olea europaea* , and *Juniperus procera* (Nyssen et al. 2009). Despite its ecological importance, the forest has experienced extensive anthropogenic degradation, leaving remnant forest patches largely confined to steep and inaccessible slopes. To date, research in the Bela Mountain area has mainly focused on land‐use and land‐cover change (Nyssen et al. 2009) and tourism potential (Teshome et al. 2023), while comprehensive assessments of woody species diversity, regeneration dynamics, and soil seed bank characteristics remain lacking.

Addressing this knowledge gap is essential for understanding the ecological processes underlying vegetation dynamics and for informing restoration and conservation initiatives in this ecologically important mountain forest. In particular, integrating aboveground vegetation characteristics with soil seed bank composition provides valuable insights into forest regeneration potential and long‐term ecosystem resilience under increasing anthropogenic and environmental pressures. Therefore, this study aimed to (i) assess woody species diversity, structural characteristics, and regeneration patterns along altitudinal gradients in the Bela Mountain Forest, and (ii) assess the species composition and density of the soil seed bank across the soil depths and altitudinal gradients.

## 2 Materials and Methods

### 2.1 Description of the Study Area

**FIGURE 1:**
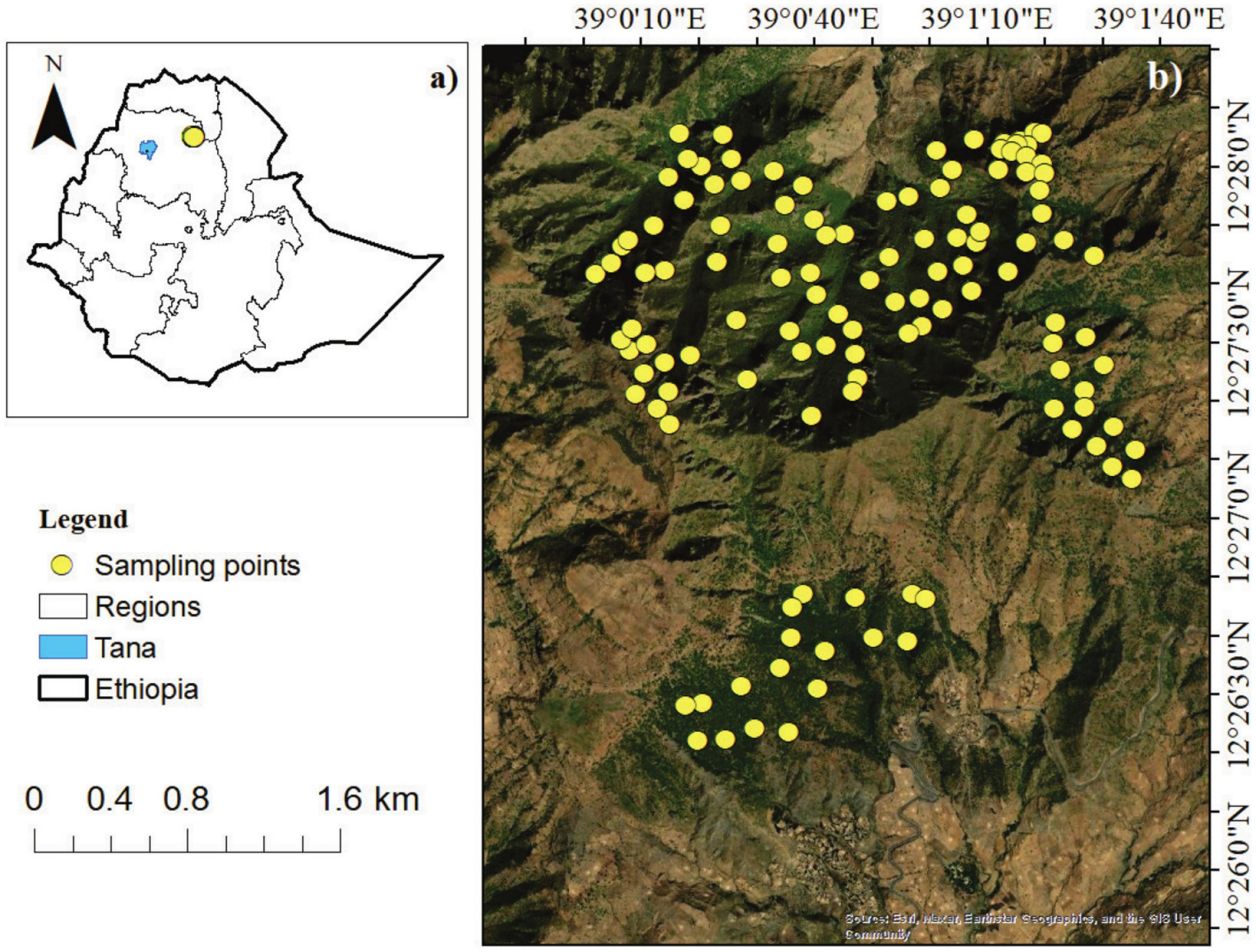
Map of the study area in the Amhara region, Ethiopia (a), Bela Mountain Forest, Gazgibla District (b).

The study was conducted in the Bela Mountain Forest, a dry Afromontane forest located in Gazgibla District, Waghimra Zone, northern Ethiopia. It is located approximately at 12°26′00″–12°28′00″N and 39°00′10′′–39°01′40′′E (Figure 1). The altitude ranges between 2700 and 3700 m above sea level. This mountain range is the source of several streams that feed into the Tekezie River and provide water for the downstream population, mainly for Woleh and Sekota towns, the Zonal city of Waghimra (Nyssen et al. 2009). The study area has a unimodal rainfall distribution pattern with a mean annual rainfall that varies from 689 to 1087 mm. The average maximum temperatures range from 12°C to 28°C.

### 2.2 Sampling and Data Collection Techniques

Before field sampling, a reconnaissance survey was conducted to assess forest conditions and identify suitable sampling locations. The field data were collected from November to December 2024. The forest boundary was delineated using a Global Positioning System (GPS). Based on altitude, the study area was stratified into three altitudinal zones: lower (2700–3100 m), middle (3101–3300 m), and upper (3301–3700 m). Six transects were established, with two transects in each altitudinal zone. The number of sample plots allocated to each zone was proportional to its forest area.

**FIGURE 2:**
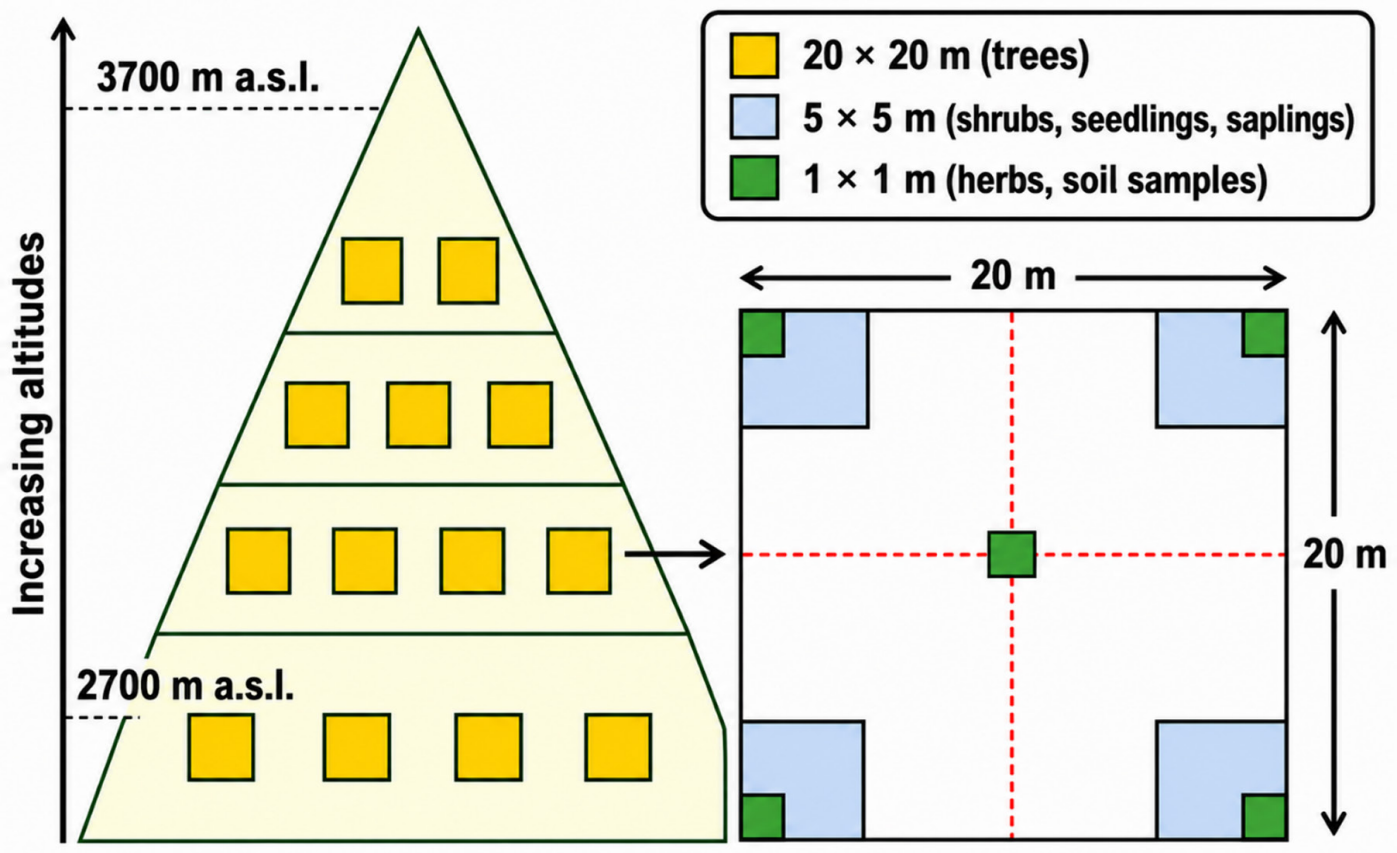
Schematic illustration of plot allocation and nested sampling design along the altitudinal gradient in the Bela Mountain Forest.

A total of 123 quadrats (20 m × 20 m) were established along the six transects, comprising 78 plots in the lower, 30 in the middle, and 15 in the upper altitudinal zones. The first plot on each transect was positioned at least 100 m from the forest edge to minimize edge effects. Subsequent plots were established at 200 m intervals along each transect, while adjacent transects were spaced 300 m apart (Figure 2).

Within each 20 m × 20 m plot, all woody individuals with a diameter at breast height (DBH, measured at 1.3 m above ground level) ≥ 2 cm and a height ≥ 2 m were measured. To assess regeneration, two 5 m × 5 m quadrats were established within each main plot to record seedlings (height < 1 m), saplings (height 1–2 m and DBH < 2 cm), and shrubs. In addition, five 1 m × 1 m quadrats were used to collect soil samples and herbaceous species (Birhanu et al. 2018). The geographic coordinates and altitude of each main plot were recorded at its center using a handheld GPS.

### 2.3 Soil Seed Bank Data Collection

Soil seed bank samples were collected from each 20 m × 20 m plot using the five‐point sampling method at three soil depths (0–5, 5–10, and 10–15 cm) (Bekele et al. 2022). The soil samples collected from the five points at each depth were thoroughly homogenized to form one composite sample per depth and transported to the Sekota Dryland Agricultural Research Center (SDARC) for germination assessment.

The soil samples were spread evenly in plastic trays and maintained under uniform moisture conditions in a screenhouse. Emerging seedlings were identified to species level whenever possible using the Flora of Ethiopia and Eritrea, field identification keys, and comparison with mature individuals recorded during the vegetation survey. After identification and counting, seedlings were removed promptly to minimize competition and prevent recounting. Seedlings that could not be reliably identified at an early stage were allowed to grow until sufficient diagnostic morphological characteristics had developed before identification. The germination experiment was conducted over 6 months (November 2024—April 2025), allowing sufficient time for seed emergence.

### 2.4 Data Analysis

To evaluate species composition in the forest, structural parameters like density, basal area, DBH, and IVI were analyzed. DBH was divided into seven size classes to examine woody species distribution: < 5 cm, 5.1–10 cm, 10.1–15 cm, 15.1–20 cm, 20.1–25 cm, 25.1–30 cm, and 30.1–35 cm (Bogale et al. 2022).

(1) $$\text{Density}=\frac{\text{Number of individuals of the species}\;}{\text{Sampled area}\;\left(\mathrm{ha}\right)}$$

Basal area (BA) was calculated using DBH measurements with the formula BA = Π(dbh^2^)/4. The importance value index (IVI) was determined by summing relative abundance (RA), relative frequency (RF), and relative dominance (RDO) (Equations 2, 3, and 4).

(2) $$\mathrm{RA}=\frac{\text{Number of individuals of}\;a\;\text{species}\;}{\text{Individuals of}\;\mathrm{all}\;\text{species in the sampled quadrats}}\times 100$$

(3) $$\mathrm{RF}=\frac{\text{number of quadrats in which}\;a\;\text{species occurred}\;}{\text{total number of sampled quadrats}}\times 100$$

(4) $$\mathrm{RDO}=\frac{\text{Species basal area}\;}{\text{Balal area of}\;\mathrm{all}\;\text{species}}\times 100$$

The species diversity was analyzed using the Shannon–Wiener diversity index (H′), and evenness was calculated in Equations 5 and 6 as follows:

(5) $$H^{\prime }=\sum_{i=1}^{s}\mathrm{Pi}\ln\mathrm{Pi}$$

(6) $$E=\frac{H^{\prime }}{H\;\max}$$

where ln: natural log; pi: proportion of the entire community made up of the ith species. E: species evenness, and H max: the number of species.

The regeneration status of dominating trees was evaluated using the proportionate distribution of individuals at seedling, sapling, and adult stages. According to Bhandari (2020) and Wani and Pant (2023), good regeneration is considered when the number of seedlings exceeds the number of saplings and mature trees. Fair regeneration is considered when the number of seedlings is less than or equal to the number of saplings, which is less than or equal to the number of mature trees. If a species only exists in tree form (no seedlings or saplings), it is termed non‐regenerating, whereas species with no trees but only seedling stages are regarded as new species. Regression analysis was also used to analyze the relationship between attitude and species diversity and regeneration status.

Before making comparisons and hypothesis testing, model residuals were evaluated using the Shapiro–Wilk test for normality and Levene's test for homogeneity of variance. A one‐way ANOVA tested variations in species diversity, richness, evenness, density, and basal area (BA) across three altitudinal gradients using R (version 4.5.1) (Team 2025). Tukey HSD tests (*p* < 0.05) compared mean differences among altitudes.

## 3 Results

### 3.1 Woody Species Composition Along Altitudinal Gradients

**FIGURE 3:**
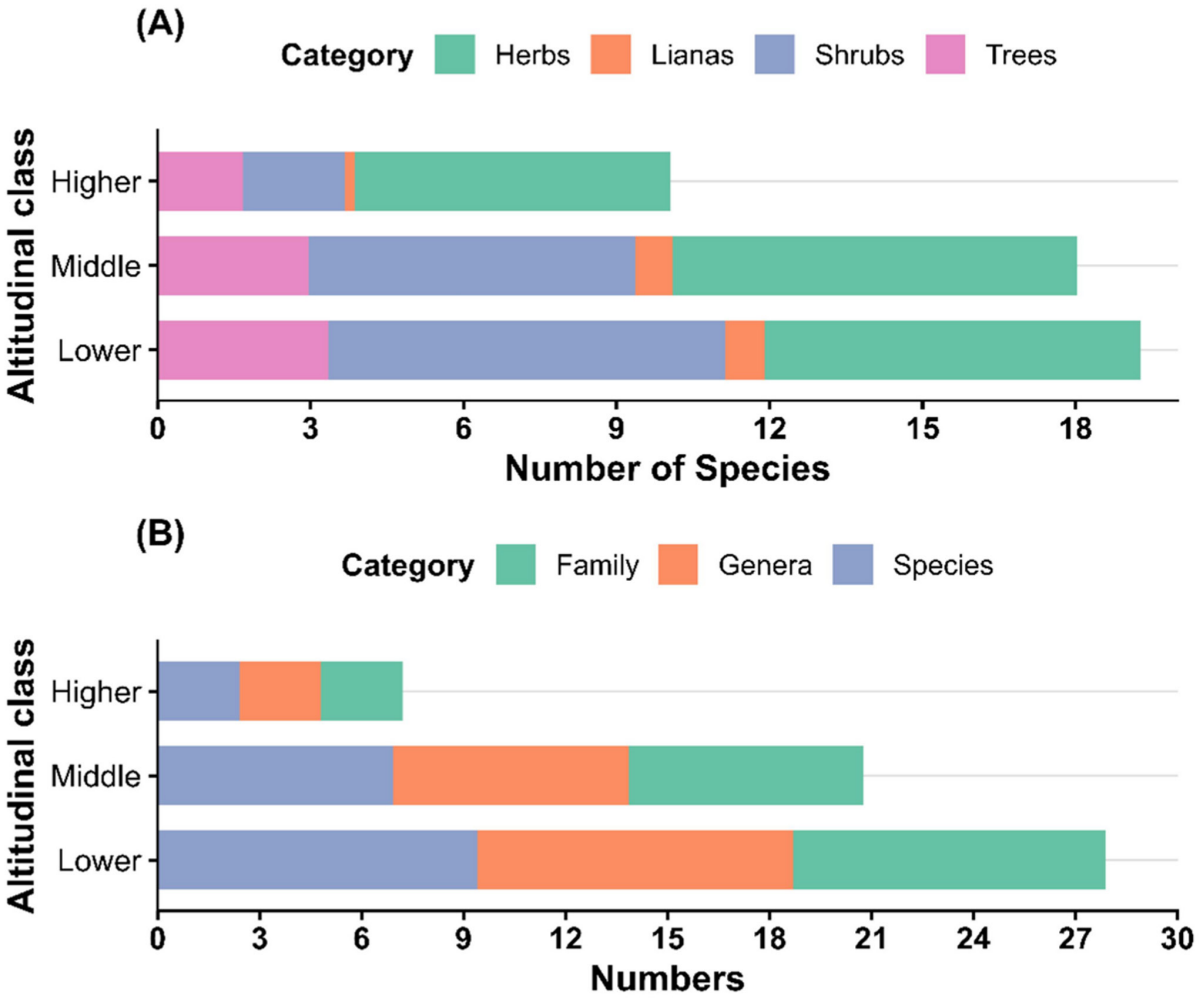
Variation in species growth habits (A) and the number of species, genera, and families (B) across altitudinal classes in Bela Mountain Forest: Lower altitude (2700–3100 m), middle altitude (3101–3300 m), and higher altitude (3301–3700 m).

The floristic analysis of the study area revealed a total of 54 woody species belonging to 31 families. From these collections, 27 species (50%) were shrubs, 23 species (42.6%) were trees, and 4 species (7.4%) were climbers (Figure 3b). There were 35, 24, and 13 species found in lower, middle, and higher altitude gradients, respectively (Figure 3b). Apocynaceae and Lamiaceae were found to be the dominant families (each with 4 species), followed by Asteraceae, Cupressaceae, Fabaceae, and Rosaceae (each with 3 species) (Figure 3b).

The proportion of climber, shrub, and tree species decreased with increasing altitudinal gradients (Figure 3a). The majority of shrub species (38.2%) were located at lower altitudes, while 33% were found at middle altitudes. Similarly, the distribution of tree species was 22% at lower altitudes, 16.2% at middle altitudes, and 15% at higher altitudes (Figure 3a). Regarding climber species, approximately 7.6% were found at lower altitudes and 7.2% at middle altitudes (Figure 3a).

### 3.2 Species Diversity and Richness Measures

**FIGURE 4:**
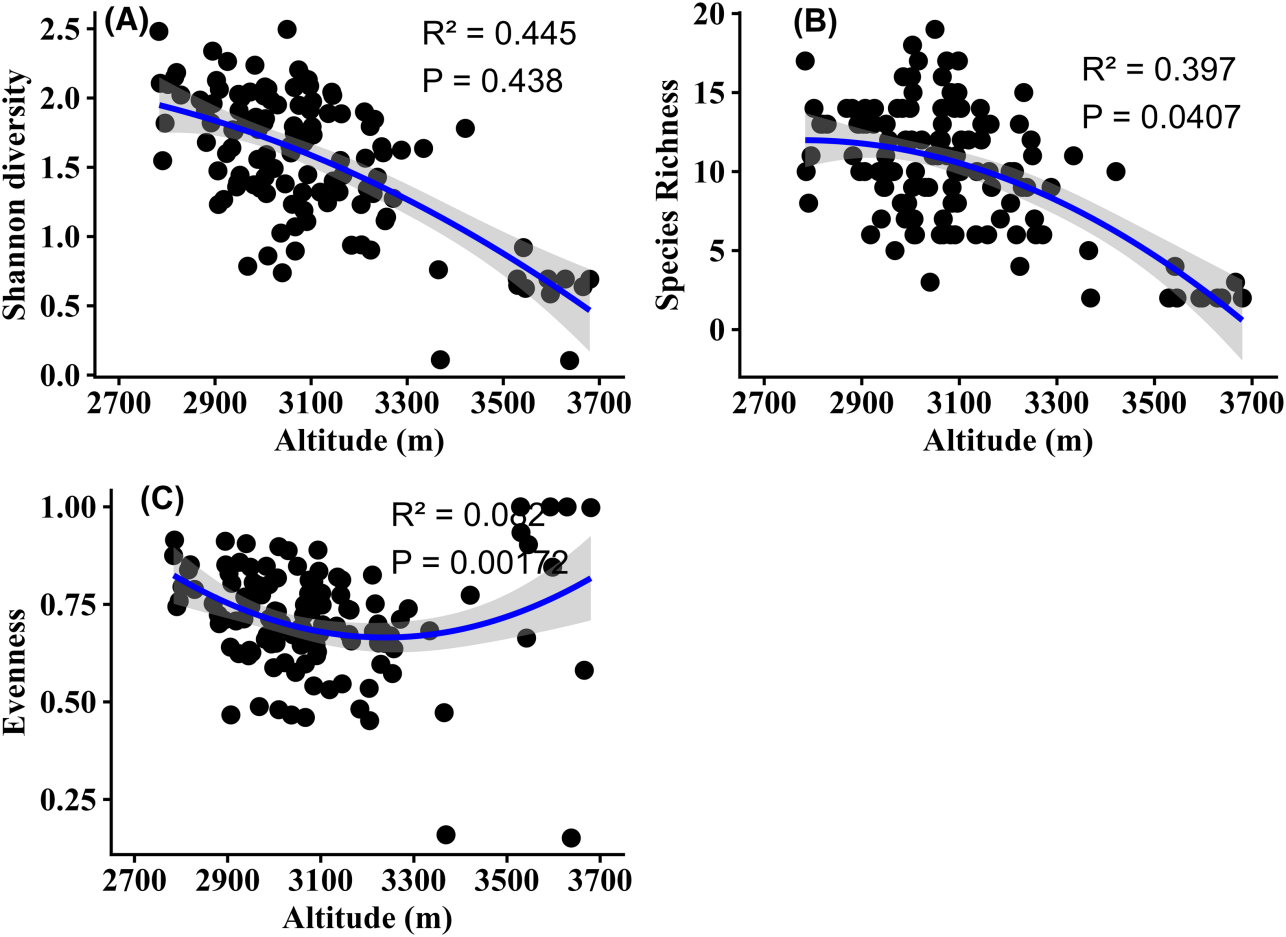
Relationships between Shannon diversity (A), species richness (B), and evenness (C) along an altitudinal gradient in the Bela Mountain Forest.

The overall Shannon diversity index and species evenness were 2.27 and 0.77, respectively. The diversity varies significantly across the altitudinal classes (lower, middle, and higher altitudes). The Shannon diversity index was highest in the lower altitude (2.37 ± 0.20), followed by the middle altitude (2.23 ± 0.19), and the lowest in the higher altitude (1.84 ± 0.28) (Table 1, Figure 4). The difference was statistically significant, with the lower altitude exhibiting significantly higher diversity than the higher altitude (*p* < 0.001). Species richness followed a similar pattern with the Shannon diversity index, with the highest mean value recorded in the lower altitude (18.9 ± 1.06), followed by the middle altitude (17.6 ± 1.27), while the higher altitude had the lowest species richness (9.87 ± 1.32). Species evenness was highest in the lower and middle altitudes, 0.80 ± 0.08 and 0.79 ± 0.08, respectively. While the lowest was found in the higher altitude (0.77 ± 0.07). However, there were no significant differences along altitudinal classes (Table 1, Figure 4).

**TABLE 1:** The Shannon diversity index, species richness, and evenness along altitudinal gradients.

Altitudinal gradients	Diversity index	Richness	Evenness	Density (ind./ha)	Basal area (m^2^/ha)
Lower	2.37 ± 0.20^a^	18.9 ± 1.06^a^	0.80 ± 0.08^a^	213 ± 26.1^b^	145 ± 12.4^a^
Middle	2.23 ± 0.19^a^	17.6 ± 1.27^a^	0.79 ± 0.08^a^	466 ± 35.5^a^	104 ± 10.2^a^
Higher	1.84 ± 0.28^b^	9.87 ± 1.32^b^	0.77 ± 0.07^a^	434 ± 21.0^a^	95.2 ± 12.1^a^

*Note:* Mean (standard error) values were calculated across altitudinal classes: lower altitude (2700–3100 m), middle altitude (3101–3300 m), and higher altitude (3301–3700 m). Different lowercase letters (a, b, c) indicate significant differences (*p* < 0.05) between altitudinal classes.

### 3.3 Species Distribution Pattern

The structural characteristics of woody species across the altitudinal gradients are presented in Table 2. At the lower altitude, *Myrsine africana* (10.78%), *Erica arborea* (10.41%), and *Vernonia bipontini* (7.06%) were the most abundant woody species recorded. In the middle altitude zone, *Erica arborea* (27.85%) showed the highest abundance, followed by *Myrsine africana* (16.89%) and *Vernonia bipontini* (10.05%). Similarly, at the higher altitude, *Myrsine africana* and *Colutea abyssinica* each accounted for 27.78% of the total woody species abundance, while *Erica arborea* contributed 19.05% (Table 2).

**TABLE 2:** Structural attributes of woody species along altitudinal gradients in Bela Mountain Forest. Values represent relative abundance (RA), relative frequency (RF), relative dominance (RDo), and importance value index (IVI).

Species	Lower altitude				Middle altitude				Higher altitude			
	RA	RF	RDo	IVI	RA	RF	RDo	IVI	RA	RF	RDo	IVI
*Acacia abyssinica*	3.4	2.6	6.1	12.0	0.0	0.0	0.0	0.0	0.0	0.0	0.0	0.0
*Allophylus abyssinicus*	1.5	0.3	4.2	6.0	0.5	0.4	8.7	9.5	0.0	0.0	0.0	0.0
*Becium grandiflorum*	2.2	1.6	0.5	4.3	0.0	0.0	0.0	0.0	0.0	0.0	0.0	0.0
*Bersama abyssinica*	0.7	0.3	1.4	2.5	0.0	0.0	0.0	0.0	0.0	0.0	0.0	0.0
*Buddleja polystachya*	0.7	0.6	1.8	3.1	0.0	0.0	0.0	0.0	0.0	0.0	0.0	0.0
*Carissa spinarum*	4.8	1.2	2.4	8.4	0.0	0.0	0.0	0.0	0.0	0.0	0.0	0.0
*Clutia abyssinica*	0.7	0.8	0.2	1.7	0.0	0.0	0.0	0.0	0.0	0.0	0.0	0.0
*Colutea abyssinica*	3.7	7.3	0.3	11.3	6.4	7.4	0.6	14.4	27.8	2.9	6.1	36.7
*Cupressus lusitanica*	4.1	1.9	8.0	13.9	0.5	4.1	2.7	7.2	0.0	0.0	0.0	0.0
*Dodonaea angustifolia*	4.1	7.1	0.7	11.9	2.7	3.3	2.6	8.6	0.0	0.0	0.0	0.0
*Erica arborea*	10.4	5.1	6.3	21.8	27.9	14.3	8.8	51	19.1	34.3	17.5	70.8
*Galiniera saxifraga*	2.6	1.1	2.3	6.0	0.9	1.6	5.0	7.5	0.0	0.0	0.0	0.0
*Hagenia abyssinica*	0.7	1.1	15.8	17.7	0.5	0.8	11.7	12.9	0.8	5.7	48.5	55.0
*Hypericum revolutum*	4.1	1.6	2.3	8.0	3.2	1.6	3.3	8.1	4.8	2.9	15.8	23.4
*Juniperus procera*	3.0	4.0	5.4	12.4	0.9	2.9	20.3	24.1	0.8	2.9	2.4	6.1
*Launaea hafunensis*	0.7	0.6	0.4	1.8	0.0	0.0	0.0	0.0	0.0	0.0	0.0	0.0
*Maytenus arbutifolia*	3.4	8.4	0.4	12.1	4.1	6.2	0.4	10.7	4.8	2.9	5.1	12.7
*Maytenus senegalensis*	0.7	0.5	2.3	3.5	0.0	0.0	0.0	0.0	0.0	0.0	0.0	0.0
*Myrica salicifolia*	0.7	1.6	12.5	14.7	1.4	4.5	13.5	19.4	0.8	8.6	19.5	28.9
*Myrsine africana*	10.8	10.1	0.3	21.2	16.9	13.1	0.6	30.6	27.8	11.4	5.7	44.9
*Nuxia congesta*	1.5	2.3	7.0	10.8	0.9	2.1	6.0	9.0	0.0	0.0	0.0	0.0
*Olea europaea*	1.9	9.9	4.8	16.6	1.8	8.2	3.5	13.5	4.8	5.7	11	21.4
*Osyris quadripartita*	1.5	5.3	0.3	7.0	1.8	5.7	0.6	8.1	4.8	5.7	5.5	15.9
*Otostegia fruticosa*	6.0	0.6	0.1	6.7	1.8	0.4	0.2	2.4	0.0	0.0	0.0	0.0
*Prunus africana*	1.5	2.0	0.9	4.4	0.0	0.0	0.0	0.0	0.0	0.0	0.0	0.0
*Rhamnus staddo*	0.7	0.5	3.1	4.3	0.0	0.0	0.0	0.0	0.0	0.0	0.0	0.0
*Rhus natalensis*	1.5	3.3	2.8	7.5	0.9	0.8	3.0	4.7	0.0	0.0	0.0	0.0
*Rhus retinorrhoea*	1.1	2.3	1.9	5.3	1.4	2.1	2.7	6.2	0.0	0.0	0.0	0.0
*Rhus retinorrhoea*	0.7	0.5	2.1	3.3	0.0	0.0	0.0	0.0	0.0	0.0	0.0	0.0
*Rosa abyssinica*	0.7	2.8	0.6	4.2	3.2	4.1	1.0	8.3	0.8	2.9	6.5	10.1
*Rumex nervosus*	1.9	3.4	0.2	5.4	5.0	1.2	0.7	7.0	1.6	2.9	5.7	10.1
*Rumex nervosus*	1.1	2.0	0.5	3.7	2.7	4.1	1.2	8.0	0.0	0.0	0.0	0.0
*Vernonia bipontini*	7.1	5.1	0.3	12.5	10.1	4.9	0.6	15.6	0.0	0.0	0.0	0.0

At the lower altitude, the most frequent species include *Myrsine africana* and *Olea europaea* , each found in 83.3% of plots. They are followed by *Maytenus arbutifolia* at 69.2%, *Colutea abyssinica* at 60.3%, and *Dodonaea angustifolia* at 58.9%. In contrast, the species *Allophylus abyssinicus*, *Rhamnus staddo*, *Maytenus senegalensis*, *Bersama abyssinica*, and *Buddleja polystachya* were the least frequent, each occurring in 3%–5% of the plots (Table 2). At the middle altitude, *Erica arborea* and *Myrsine africana* were the most frequently occurring species, each being recorded in all sampled plots (100% frequency). These were followed by *Olea europaea* and *Colutea abyssinica*, which occurred in 66.7% and 60% of the plots, respectively. In contrast, *Hagenia abyssinica*, *Rhus natalensis*, and *Juniperus procera* were among the least frequently recorded species in this altitudinal zone. At the higher altitude, *Erica arborea* was the most frequently occurring species, being recorded in 80% of the sampled plots. In contrast, the remaining species showed relatively low frequencies, occurring in only 6%–26% of the plots (Table 2).

Based on the IVI value, *Erica arborea* (21.80), *Myrsine africana* (21.15), *Olea europaea* (16.55), and *Hagenia abyssinica* (17.65) were the most ecologically important species at lower altitudes. In the middle altitude, *Erica arborea* (50.98), *Myrsine africana* (30.59), *Juniperus procera* (24.11), and *Myrica salicifolia* (19.41) were also the most important species. Furthermore, *Erica arborea* (70.78), *Hagenia abyssinica* (54.97), *Myrsine africana* (44.92), and *Colutea abyssinica* (36.94) were the most ecologically important species at the higher altitude (Table 2).

### 3.4 Regeneration Pattern

**FIGURE 5:**
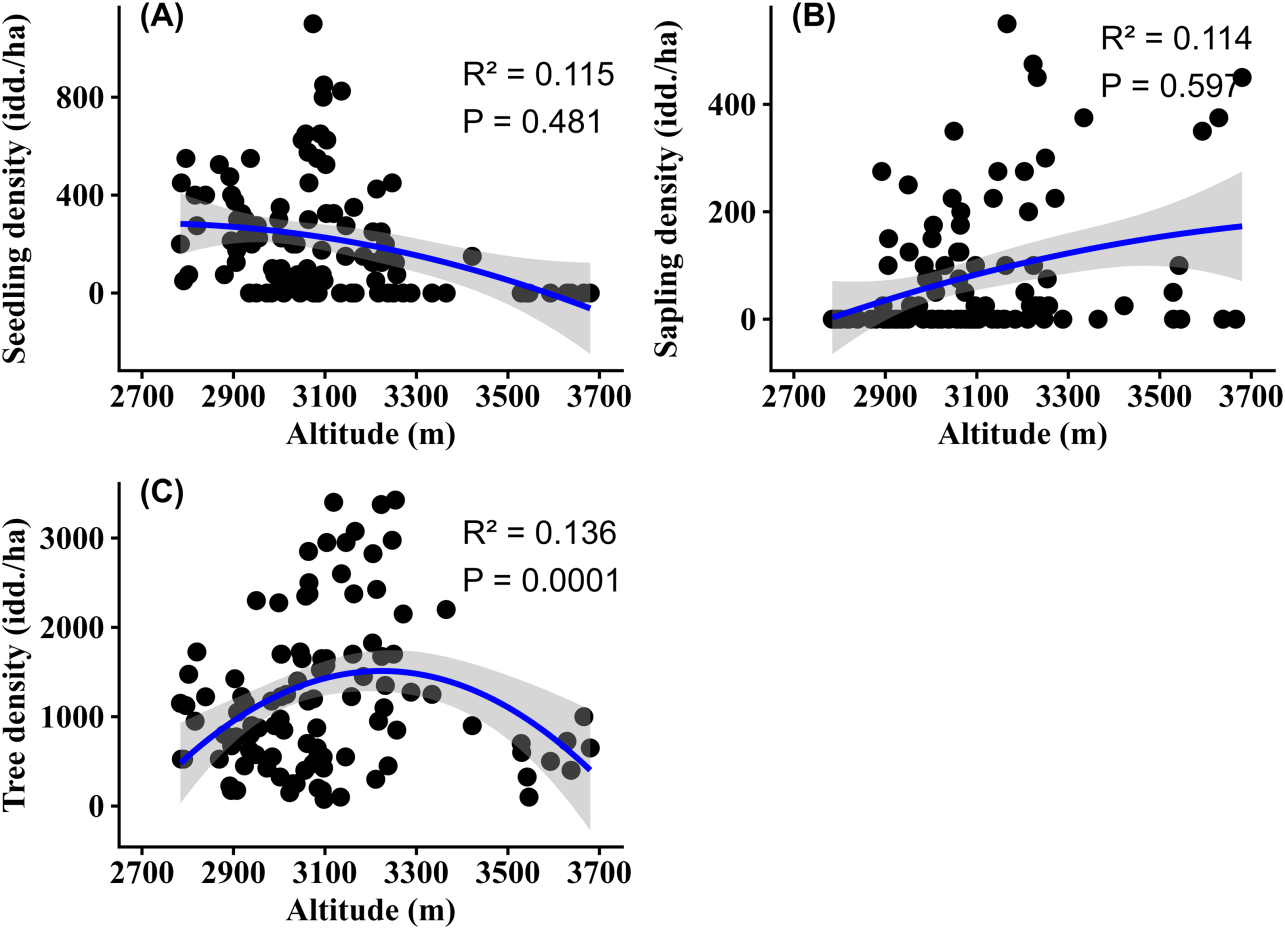
Relationships between seedling density (A), sapling density (B), and tree density (C) along an altitudinal gradient in the Bela Mountain Forest.

The overall seedling density along the altitudinal gradient ranged from 250 to 27,750 ind./ha (Figure 5). The highest seedling density (27,750 ind./ha) was recorded at the lower altitude, whereas the lowest density (250 ind./ha) occurred at the higher altitude (Figure 5a). Similarly, sapling density varied from 475 to 1575 ind./ha along the altitudinal gradients (Figure 5b). The regeneration status of woody species was predominantly classified as fair, representing 36.4%, 35.7%, and 50% of the species at the lower, middle, and higher altitudes, respectively (Appendix 2). Species with no regeneration accounted for 31.8% and 33.3% of the total woody species at the lower and higher altitudes, respectively. In addition, 18.2% of the woody species recorded at the lower altitude were identified as newly regenerating species (Appendix 2).

The regeneration status of *Erica arborea* , *Dodonaea angustifolia*, *Olea europaea* , and *Juniperus procera* was categorized as fair at both the lower and middle altitudes (Appendix 2). At the lower altitude, *Carissa spinarum*, *Maytenus senegalensis*, *Hagenia abyssinica*, *Acacia abyssinica* , and *Allophylus abyssinicus* showed no regeneration. Similarly, *Nuxia congesta*, *Rhus retinorrhoea*, and *Allophylus abyssinicus* did not regenerate at the middle altitude, whereas *Hagenia abyssinica* and *Myrica salicifolia* showed no regeneration at the higher altitude. Furthermore, *Nuxia congesta*, *Bersama abyssinica*, *Acokanthera schimperi*, and *Rhus retinorrhoea* were identified as newly regenerating species at the lower altitude; *Prunus africana* and *Rhus retinorrhoea* at the middle altitude; and *Juniperus procera* at the higher altitude (Appendix 2).

### 3.5 Population Structure

**FIGURE 6:**
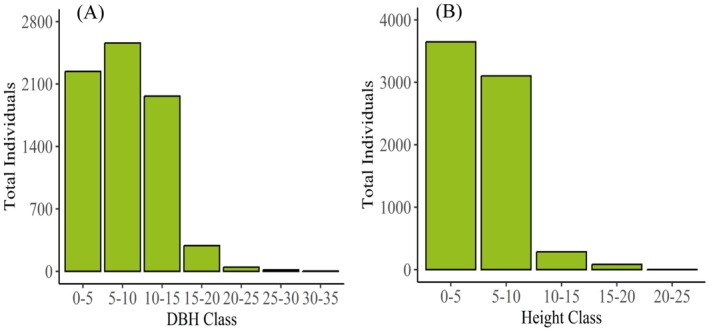
Diameter at breast height (DBH) class distribution (a) and height class distribution (b) of woody species in the Bela Mountain Forest.

The distribution of the DBH classes revealed that the second class contained the majority of woody species, followed by the first and third classes (Figure 6a). The second class recorded around 36% (2560 ind./ha) of the total population, whereas the first and third classes accounted for 31.6% (2242 ind./ha) and 27.7% (1965 ind./ha), respectively. In contrast, the seventh, sixth, and fifth DBH classes had the fewest individuals, accounting for 0.04%, 0.24%, and 0.66% of the overall population, respectively (Figure 6a). This distribution implies that the study forest did not regenerate in a typical inverted J pattern. Furthermore, the majority of woody species were concentrated in the first height class, accounting for 3649 ind./ha. Overall, the height class distribution of woody species exhibited an inverted J‐shaped pattern (Figure 6b).

**FIGURE 7:**
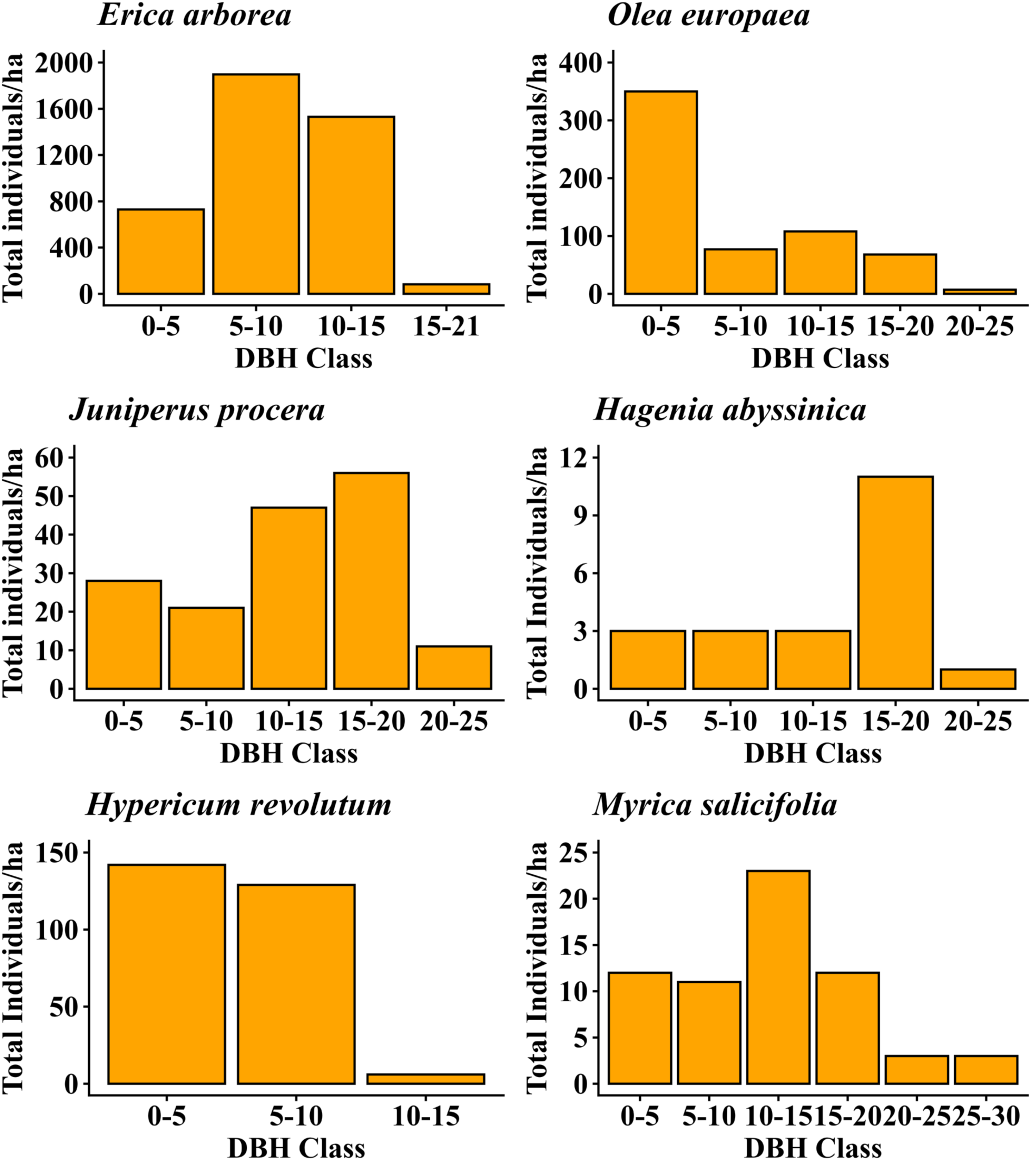
Diameter at breast height (DBH) class distribution of selected woody species in the Bela Mountain Forest.

Among the six chosen woody species, the majority displayed unusual population structures (Figure 7). The analysis of population structures using DBH class distribution revealed two distinct patterns, indicating different population dynamics among the species (Figure 7). The first pattern, characterized by a bell shape, showed a relatively high number of individuals in the middle DBH classes, with a gradual decline in the lower and upper classes. This pattern was observed in species such as *Erica arborea* , *Juniperus procera*, *Myrica salicifolia*, and *Hagenia abyssinica* (Figure 7). In contrast, *Olea europaea* and *Hypericum revolutum* showed the second pattern, an inverted J‐shape, with a high density of individuals in the lower DBH classes and a progressive tapering off in the upper classes (Figure 7).

### 3.6 Density and Basal Area of Woody Species

In the Bela Mountain Forest, the overall density of woody species with a DBH of ≥ 2.5 cm was 3434 ind./ha (Table 1). The most numerous species included *Erica arborea* (726 ind./ha), *Cupressus lusitanica* (482 ind./ha), *Carissa spinarum* (319 ind./ha), and *Hypericum revolutum* (315 ind./ha). In contrast, the least dense species were *Maytenus senegalensis* (33 ind./ha), *Eucalyptus globulus* (37 ind./ha), *Hagenia abyssinica* (47 ind./ha), *Buddleja polystachya* (44 ind./ha), *Acokanthera schimperi* (50 ind./ha), and *Bersama abyssinica* (50 ind./ha) (Table 1).

A statistical analysis of density across each altitudinal gradient reveals significant variations (*p* < 0.05) across the three altitudes (Table 1). The medium altitude had the highest density (466 ± 35.5 ind./ha), followed by the upper altitude (434 ± 21 ind./ha) and the lower altitude (213 ± 26.1 ind./ha) (Table 1).

The overall basal area (with a DBH of ≥ 2.5 cm) was 149.4 m^2^/ha (Table 1). *Eucalyptus globulus* (702.8 m^2^/ha), *Hagenia abyssinica* (286.2 m^2^/ha), *Cupressus lusitanica* (246.7 m^2^/ha), *Myrica salicifolia* (222.5 m^2^/ha), *Juniperus procera* (155.9 m^2^/ha), *Olea europaea* (136.8 m^2^/ha), and *Acacia abyssinica* (114.9 m^2^/ha) contributed significantly to this total (Table 1). The remaining 12 species provided 913.6 m^2^/ha, or 32.8% of the total basal area in the Bela forest. The statistical analysis of basal area over three altitudinal gradients indicates no significant variations (*p* < 0.05) (Table 1). The average basal area at lower, middle, and higher altitudes was 145 m^2^/ha, 104 m^2^/ha, and 95.2 m^2^/ha, respectively (Table 1).

### 3.7 Species Composition and Seedling Density in the Soil Seed Bank

A total of 68 species were recorded in the soil seed bank collected in the study area (Table 3). Herbs constituted the largest category, with 42 species (61.8%), followed by shrubs with 18 species (26.5%), trees with 4 species (6%), and climbers with 4 species (6%) (Table 3). Among the woody species identified in the soil seed bank were *Erica arborea* , *Myrsine africana*, and *Clutia lanceolata*. Species richness varied along altitudinal gradients, ranging from 37 to 48 species. The highest species composition was observed at middle altitudes, with 48 species, followed by lower altitudes with 39 species and higher altitudes with 37 species (Table 3).

**TABLE 3:** Seedling density (Ind./m^2^) distribution across altitudinal classes and soil depths in the Bela Mountain Forest.

Altitudinal range	Total density	%	Soil depths					
			0–5 cm	%	5–10 cm	%	10–15 cm	%
Lower	1813	12.0	1406	14.1	264	7.4	143	8.8
Middle	5072	33.5	3281	32.9	1338	37.7	453	27.9
Higher	8256	54.5	5279	53.0	1948	54.9	1029	63.3
Total	15,141	100	9966	65.8	3550	23.5	1625	10.7

The total seedling density across all altitudinal classes and soil depths was 15,141 seedlings/m^2^ (Table 3). Seedling density consistently increased with increasing altitude across all soil depths. The highest seedling density was recorded at the higher altitude (8256 seedlings/m^2^), followed by the middle altitude (5072 seedlings/m^2^) and the lower altitude (1813 seedlings/m^2^) (Table 3). In all altitudinal gradients, overall species density was inversely proportional to soil depth (Table 3). The upper layer had the highest species density (9966 seedlings/m^2^), followed by the second layer (3550 seedlings/m^2^) and the third layer (1625 seedlings/m^2^) (Table 3).

## 4 Discussion

### 4.1 Species Diversity and Richness

Altitudinal variation in the Bela Mountain Forest significantly influenced species diversity and richness. Previous studies have demonstrated that altitude strongly affects vegetation structure, floristic composition, and species diversity in mountainous ecosystems (Zhang et al. 2024; Gillani et al. 2025). In Ethiopia, altitude is recognized as one of the major ecological factors determining floristic composition and plant community distribution (Woldu et al. 2020; Yimer and Atsbha 2025). Variations in altitude are often associated with changes in climatic conditions, soil properties, and ecological processes, which collectively influence species establishment and dominance patterns (Song et al. 2021; Qianwen et al. 2022).

The present study revealed significant differences (*p* < 0.05) in species diversity and richness among the lower, middle, and higher altitudinal classes. Specifically, species richness and Shannon diversity were markedly lower at the higher altitude compared with the lower and middle altitudes. The reduced diversity and richness observed at higher elevations may be associated with harsher environmental conditions, including lower temperatures, shallow and less fertile soils, and reduced water availability (Wani et al. 2023; Zhang et al. 2024). Such environmental constraints can limit species establishment, growth, and survival, thereby reducing overall species diversity.

In contrast, no significant differences were observed between the lower and middle altitudinal classes in terms of species diversity and richness. This finding is consistent with earlier studies that reported a decline in species diversity and richness with increasing altitude (Gebrehiwot et al. 2019; Wani et al. 2023; Negi et al. 2024; Borago 2025). However, other studies have documented increasing species diversity with altitude (Woldu et al. 2020; Bogale et al. 2023; Yimer and Atsbha 2025) or a hump‐shaped relationship between diversity and elevation (Gebrehiwot et al. 2019; He et al. 2023). These inconsistencies among studies may arise from differences in climatic conditions, topography, disturbance regimes, sampling methods, and local environmental heterogeneity across study areas. The decline in species diversity and richness at higher altitudes in the present study may therefore be attributed to reduced nutrient availability, limited microclimatic heterogeneity, and increasing environmental stress associated with elevation.

### 4.2 Density and Basal Area of Woody Species

The Bela Mountain Forest had a total woody density of 100 to 4100 stems/ha and a basal area ranging from 54.8 to 76.3 m^2^/ha. This variation is due to microclimate, stand structure, and human disturbance regime (Yirga et al. 2019). This is higher than other studies in dry Afromontane forests, for example, Zengena forest (2202 ind./ha) (Tadele et al. 2014), Gelawdiwos forest (2016 ind./ha) (Mucheye and Yemata 2020), Endrias forest (3253.5 ind./ha) (Tebabal et al. 2024), and Amoro forest (2860.5 ind./ha) (Birhanu et al. 2018). However, it is lower than Harego forest (4400.8 ind./ha) (Bogale et al. 2022) and Kumuli Forest (7791 ind./ha) (Woldemariam et al. 2016). These variations might be due to the level of anthropogenic disturbance (Yirga et al. 2019; Legese et al. 2025) and differences in topographic gradients and habitat preferences in the forest (Wani et al. 2023; Abrha et al. 2024). The density along the three altitudinal gradients varied significantly. The lower altitude showed a much lower density (213 ind./ha) compared to the intermediate and upper altitudes. This is primarily due to increased human activity, such as tree cutting and grazing operations, which occur at lower altitudes.

### 4.3 Regeneration Status

Natural regeneration is a vital process for ensuring the resilience of forests and the emergence of a younger generation of trees (Bhandari 2020). Forest regeneration capacity is determined by the presence of a larger density of seedlings, saplings, and mature trees, which might vary depending on forest structure and physical circumstances (Negi et al. 2024; Gillani et al. 2025). We studied forest health across three altitudinal classes by analyzing the regeneration status of tree species, namely seedling, sapling, and adult tree density. The regeneration status of woody species in the Bela Mountain Forest varied significantly along the altitudinal gradient, with various species recruiting and establishing under different environmental conditions. The higher seedling density was observed at the lower altitude and the lowest at the higher altitudes. These are attributed to relatively warmer temperatures, better nutrient availability, and deeper soils that help with seed germination and seedling establishment. Furthermore, the differences in sapling density along altitude may be due to the variability of the successful transition of seedlings to later growth stages.

The lower and middle altitudes exhibited variability in regeneration patterns, with different species such as *Erica arborea* , *Dodonaea angustifolia*, *Olea europaea* , *Cupressus lusitanica* , and *Juniperus procera* having higher seedling density, demonstrating their ecological adaptability across different altitudinal conditions. These species may possess broader ecological tolerance and higher resilience to environmental stress compared to poorly regenerating species. However, the relatively high proportion of species exhibiting no regeneration across altitudes, which may be associated with poor seed viability, limited dispersal capacity, anthropogenic disturbances, and limited soil seed bank potential of the forest (Yirga et al. 2019; Bogale et al. 2023; Legese et al. 2025). In contrast, species categorized as newly regenerating, including *Nuxia congesta*, *Prunus africana* , and *Juniperus procera*, may represent species responding to recent environmental changes or favorable microsite conditions for recruitment. Overall, the findings indicate that altitude strongly influences woody species regeneration dynamics in the Bela Mountain Forest through its effects on microclimate, soil conditions, and disturbance intensity.

### 4.4 Soil Seed Bank Composition and Regeneration Potential

Soil seed banks serve as reservoirs of seeds that can regenerate when environmental conditions become favorable (Zhang et al. 2021). In the Bela Mountain forest, the soil seed bank analysis revealed 68 species, with herbaceous species predominating at 62.5%, outnumbering woody species. This prevalence of herbs may be linked to the disturbance levels present in the area (Bekele et al. 2022; Girmay et al. 2022). Furthermore, herbaceous species exhibit resilience under challenging conditions, allowing them to reintegrate into the ecosystem without the need for reintroduction (Durak et al. 2022). Additionally, Savadogo et al. (2017) noted that grasses and herbs typically possess smaller seeds compared to many woody species. This smaller seed size enhances their chances of being buried in deeper soil layers, which aligns with the findings of the current study. The concept is further reinforced by the fact that pioneer species tend to produce a large number of seeds, contributing to the high density of small seeds found in soil seed banks (Ekasari et al. 2021).

## 5 Conclusion and Recommendations

This study documented 54 woody species belonging to 31 families in the Bela Mountain Forest, demonstrating the ecological importance of this montane ecosystem. Woody species diversity, composition, and regeneration varied significantly along the altitudinal gradient, with lower and middle elevations supporting higher diversity and better regeneration than upper altitudes. These findings highlight the influence of altitude on vegetation patterns and identify lower and middle altitudinal zones as priority areas for biodiversity conservation and sustainable forest management. The soil seed bank contained 68 plant species, dominated by herbaceous species (62.5%) across both soil depths and altitudinal gradients, whereas woody species were poorly represented. This indicates that the existing soil seed bank has limited potential to facilitate the natural regeneration of woody vegetation in degraded areas. Consequently, restoration efforts should not rely solely on the soil seed bank but should incorporate active restoration measures, including enrichment planting with native woody species, nursery‐raised seedlings, direct seeding where appropriate, and effective control of anthropogenic disturbances. Integrating these approaches with the conservation of remaining forest patches will strengthen ecosystem resilience and support the long‐term restoration and sustainable management of the Bela Mountain Forest.

## Author Contributions

**Abebe Shumie:** conceptualization (lead), data curation (equal), formal analysis (lead), investigation (equal), methodology (equal), software (lead), writing – original draft (lead). **Yekoye Alene:** data curation (equal), investigation (equal), methodology (equal), writing – review and editing (equal). **Geta Mesegan:** data curation (equal), methodology (equal), writing – review and editing (equal). **Sileshi Asmare:** data curation (equal), investigation (equal), validation (supporting), writing – review and editing (supporting). **Getu Abebe:** formal analysis (supporting), supervision (equal), validation (equal), writing – review and editing (equal). **Mubarek Eshetie:** formal analysis (supporting), supervision (supporting), validation (equal), writing – review and editing (equal). **Menale Wondie:** conceptualization (supporting), data curation (supporting), methodology (supporting), supervision (lead), writing – review and editing (equal).

## Funding

The research was conducted with financial support from the Sekota Dryland Agricultural Research Center (SDARC).

## Conflicts of Interest

The authors declare no conflicts of interest.

## Supporting information


**Data S1:** ece374102‐sup‐0001‐Supinfo.zip.

## Data Availability

All the required data are uploaded as supporting information (Data S1).

## Appendix 1 List of the Collected Woody Species in the Bela Mountain Forest, Including Their Vernacular Name, Family, and Lifeform

**Table:**

Scientific Name	Family	Local name	Habit
*Acacia abyssinica*	Fabaceae	Bazira girar	Tree
*Acacia saligna*	Fabaceae	Saligna grar	Tree
*Acokanthera schimperi*	Apocynaceae	Merez	Tree
*Allophylus abyssinicus*	Sapindaceae	Embuf	Tree
*Asparagus africanus*	Asparagaceae	Yeset kest	Climber
*Becium grandiflorum*	Lamiaceae	Mentesie	Tree
*Bersama abyssinica*	Melianthaceae	Azamir	Tree
*Buddleja polystachya*	Scrophulariaceae	Anfar	Tree
*Carissa spinarum*	Apocynaceae	Agam	Tree
*Ceropegia sankuruensis*	Apocynaceae	Sukika	Climber
*Clematis simensis*	Ranunculaceae	Azo hareg	Climber
*Clutia abyssinica*	Peraceae/Euphorbiaceae	Duaduate	Shrub
*Colutea abyssinica*	Fabaceae	Fyele fej	Shrub
*Cupressus lusitanica*	Cupressaceae	Yefernj tsid	Tree
*Dodonaea angustifolia*	Sapindaceae	Kitkita	Shrub
*Dombeya torrida*	Malvaceae	Wolkf	Tree
*Echinops macrochaetus*	Asteraceae	Yedega Kushele	Shrub
*Ekebergia capensis*	Meliaceae	Lol	Tree
*Erica arborea*	Ericaceae	Asta	Tree
*Eucalyptus globulus*	Myrtaceae	Nech bahirzaf	Tree
*Galiniera saxifraga*	Rubiaceae	Tota kolet	Tree
*Hagenia abyssinica*	Rosaceae	Koso	Tree
*Hypericum revolutum*	Hypericaceae	Muja	Tree
*Jasminum grandiflorum*	Oleaceae	Tembelel	Climber
*Juniperus procera*	Cupressaceae	Yehabesha tsid	Tree
*Launaea hafunensis*	Asteraceae	Ageswe	Shrub
*Leonotis ocymifolia*	Lamiaceae	Yeferes zeng	Shrub
*Lippia adoensis*	Verbenaceae	Keskese	Shrub
*Maytenus arbutifolia*	Celastraceae	Atat	Shrub
*Maytenus senegalensis*	Celastraceae	Kokoba	Tree
*Mukia maderaspatana*	Cucurbitaceae	Nech hareg	Climber
*Myrica salicifolia*	Myricaceae	Shnet	Tree
*Myrsine africana*	Primulaceae	Qechemo	Shrub
*Nuxia congesta*	Stilbaceae	Askuar	Tree
*Olea europaea*	Oleaceae	Weira	Tree
*Osyris quadripartita*	Santalaceae	Keret	Shrub
*Otostegia fruticosa*	Lamiaceae	Nechilo	Shrub
*Otostegia integrifolia*	Lamiaceae	Geram tinjut	Shrub
*Prunus africana*	Rosaceae	Tikur enchet	Tree
*Rhamnus prinoides*	Rhamnaceae	Gesho	Shrub
*Rhamnus staddo*	Rhamnaceae	Todo	Tree
*Rhus natalensis*	Anacardiaceae	Talo	Tree
*Rhus retinorrhoea*	Anacardiaceae	Dabo talo	Tree
*Rosa abyssinica*	Rosaceae	Qega	Shrub
*Rumex nervosus*	Polygonaceae	Embacho	Shrub
*Solanum anguivi*	Solanaceae	Yewusha embay	Shrub
*Solanum marginatum*	Solanaceae	Yedega embay	Shrub
*Stephania abyssinica*	Menispermaceae	Yeayt hareg	Climber
*Urtica hypsoleucodendron*	Urticaceae	Toro hareg	Climber
*Vernonia bipontini*	Asteraceae	Tija lebek	Shrub

## Appendix 2 Regeneration Pattern of Tree Species Along Altitudinal Gradients

**Table:**

Altitudinal Class	Altitude range (m)	Species	Seedling (ind./ha)	Sapling (ind./ha)	Trees (ind./ha)	Regeneration status
Lower	2700–3100	*Carissa spinarum*	0	0	2550	No
		*Buddleja polystachya*	25	0	1300	Poor
		*Nuxia congesta*	50	0	0	New
		*Erica arborea*	5187	50	25,000	Good
		*Bersama abyssinica*	0	0	100	New
		*Rhus retinorrhoea*	125	25	1200	Fair
		*Dodonaea angustifolia*	6675	175	11,000	Good
		*Maytenus senegalensis*	0	0	100	No
		*Hagenia abyssinica*	0	0	375	No
		*Ekebergia capensis*	112	25	0	Good
		*Acokanthera schimperi*	50	0	0	New
		*Hypericum revolutum*	2400	75	3025	Good
		*Acacia abyssinica*	0	0	3800	No
		*Prunus africana*	1300	25	375	Good
		*Allophylus abyssinicus*	0	0	300	No
		*Rhamnus staddo*	0	0	100	No
		*Galiniera saxifraga*	0	225	1275	Poor
		*Olea europaea*	5050	775	6450	Good
		*Rhus natalensis*	525	0	0	New
		*Cupressus lusitanica*	5575	50	3025	Good
		*Juniperus procera*	600	25	3225	Good
Middle	3101–3300	*Nuxia congesta*	0	0	200	No
		*Erica arborea*	3125	1125	56,650	Good
		*Rhus retinorrhoea*	0	0	75	No
		*Dodonaea angustifolia*	100	0	925	Fair
		*Hagenia abyssinica*	0	250	25	Poor
		*Hypericum revolutum*	0	250	925	Poor
		*Myrica salicifolia*	150	25	575	Good
		*Prunus africana*	200	0	0	New
		*Allophylus abyssinicus*	0	0	25	No
		*Galiniera saxifraga*	0	25	75	Poor
		*Olea europaea*	1975	100	775	Good
		*Rhus natalensis*	25	0	0	New
		*Cupressus lusitanica*	0	0	25	No
		*Juniperus procera*	25	25	150	Fair
Higher	3301–3700	*Erica arborea*	75	475	14,325	Fair
		*Hagenia abyssinica*	0	0	100	No
		*Hypericum revolutum*	100	0	150	Fair
		*Myrica salicifolia*	0	0	250	No
		*Olea europaea*	25	0	75	Fair
		*Juniperus procera*	25	0	0	New
